# Construction of MXene-Based Heterostructured Hybrid Separators for Lithium–Sulfur Batteries

**DOI:** 10.3390/molecules30081833

**Published:** 2025-04-19

**Authors:** Xiao Zhang, Guijie Jin, Min Mao, Zirui Wang, Tianyu Xu, Tongtao Wan, Jinsheng Zhao

**Affiliations:** 1 Shandong Provincial Key Laboratory of Chemical Energy Storage and Novel Cell Technology, School of Chemistry and Chemical Engineering, Liaocheng University, Liaocheng 252059, China; xiaozhang@lcu.edu.cn (X.Z.); jguijie@163.com (G.J.); zruiwang2025@163.com (Z.W.); 15506352558@163.com (T.X.); 2 Hubei Key Laboratory of Automotive Power Train and Electronic Control, School of Automotive Engineering, Hubei University of Automotive Technology, Shiyan 442002, China; 20230056@huat.edu.cn

**Keywords:** MXenes, heterostructures, separators, shuttle effect, redox kinetics, lithium–sulfur batteries

## Abstract

The advancement of lithium–sulfur (Li-S) batteries has been hindered by the shuttle effect of lithium polysulfides (LiPSs) and sluggish redox kinetics. The engineering of functional hybrid separators is a relatively simple and effective coping strategy. Layered transition-metal carbides, nitrides, and carbonitrides, a class of emerging two-dimensional materials termed MXenes, have gained popularity as catalytic materials for Li-S batteries due to their metallic conductivity, tunable surface chemistry, and terminal groups. Nonetheless, the self-stacking flaws and easy oxidation of MXenes pose disadvantages, and developing MXene-based heterostructures is anticipated to circumvent these issues and yield other remarkable physicochemical characteristics. Herein, recent advances in the construction of MXene-based heterostructured hybrid separators for improving the performance of Li-S batteries are reviewed. The diverse conformational forms of heterostructures and their constitutive relationships with LiPS conversion are discussed, and the general principles of MXene surface chemistry alterations and heterostructure designs for enhancing electrochemical performance are summarized. Lastly, tangible challenges are addressed, and advisable insights for future research are shared. This review aims to highlight the immense superiority of MXene-based heterostructures in Li-S battery separator modification and inspire researchers.

## 1. Introduction

In recent years, the wide application of portable electronic products, electric vehicles, and energy storage stations has greatly improved the convenience of our daily lives. The rapid development of these storage devices has given rise to huge demand and extensive research on energy storage systems with a high capacity and energy density. With a theoretical specific capacity of 1675 mAh g^−1^ and an energy density of 2600 Wh kg^−1^, which is 3–5 times higher than that of conventional lithium-ion batteries, lithium–sulfur (Li-S) batteries have the advantages of cost-effectiveness, non-toxicity, and a sulfur-rich content and are considered one of the most promising next-generation secondary storage batteries [1,2,3]. Due to the complex reactions during the charge/discharge process of Li-S batteries, there are still considerable challenges in practical applications. The main reason is the shuttle effect of soluble long-chain lithium polysulfides (LiPSs) (Li_2_S_x_; 4 ≤ x ≤ 8) [4,5]. This is because LiPSs move between the cathode and anode during charging/discharging, resulting in the loss of active materials and the decay of battery capacity. It should be emphasized that the shuttling effect is a surface phenomenon, while the reaction kinetics are the root cause. Slow reaction kinetics, stemming from multi-step sulfur redox reactions, inevitably lead to poor cycling and rate performance [6,7]. In addition, the formation of “lithium dendrites” [8] also hinders the practicality of Li-S batteries.

In order to solve the above problems and realize high-performance Li-S batteries, researchers have carried out extensive research involving electrode structure design, novel electrolytes, and functional separators [9,10,11]. A functional separator is a relatively convenient and effective method for achieving high sulfur utilization and long lifespans in Li-S batteries [12,13]. Polypropylene (PP) separators are widely used in commercial lithium batteries, but they cannot sufficiently inhibit the shuttle effect [14] due to their macroporous nature. Modifying PP separators with various functional materials, such as metal oxides [15], carbides [16], nitrides [17], borides [18], and sulfides [19], can not only prevent the migration of polysulfides through physical/chemical interactions with LiPSs but also effectively promote redox kinetics, achieving high-performance Li-S batteries [20].

Layered transition-metal carbides, nitrides, and carbonitrides, a class of new two-dimensional (2D) materials called MXenes, have attracted much attention in recent years due to their excellent physical and chemical properties [21,22,23]. The general formula of the MXene family is M_n+1_X_n_T_x_ (*n* = 1–3), where M represents the transition metal (such as Ti, Mo, V, etc.), X stands for the carbide or nitride group (C or N) [24,25], and T_x_ refers to the terminations on the surface [25,26,27]. Compared with traditional materials such as carbon, metal compounds, and polymers (Table 1), MXenes exhibit infinite potential for regulating lithium and sulfur conversion in Li-S batteries due to key characteristics such as tunable terminations, high electrical conductivity, abundant defects and vacancies, a large specific surface area, and good mechanical strength, controlled by different chemical compositions and terminating functional groups on their surfaces [28,29]. Nevertheless, hydrogen bonds and van der Waals forces within the layers make MXene nanosheets likely to self-restack, limiting the rapid transport of ions and the effective exposure of active sites [30]. Additionally, the easily oxidized nature of MXenes is also a shortcoming that cannot be ignored. By rationally constructing MXene-based heterostructured hybrid separators, on the one hand, the built-in electric field spontaneously generated by the redistribution of electrons at the heterointerfaces can accelerate the migration of charge carriers and the anchoring of polysulfides, and more active sites can be induced through the adjustment of the coordination environment [31,32]. On the other hand, this structure can bypass the drawbacks of MXenes and acquire other amazing physicochemical properties, thereby synergistically enhancing the performance of Li-S batteries (Table 1).

In this review, the research progress on MXene-based heterostructured hybrid separators for Li-S batteries is summarized. Different conformational forms of the heterostructures and their intrinsic relationship with the conversion of LiPSs are discussed. The fundamental guidelines for enhancing electrochemical performance by adjusting the surface chemistry of MXenes and designing heterostructures are outlined. At the end of this review, the prospects and challenges in this field are looked ahead to, and a few perspectives are shared.

## 2. Overview of MXenes and MXene-Based Heterostructures

### 2.1. Overview of MXenes

**Figure 1:**
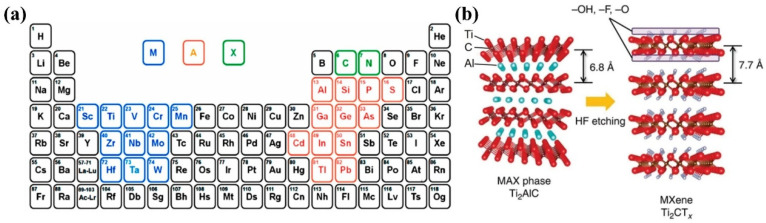
(**a**) Elements of MAX phase formation [33] (copyright 2024, ELSEVIER SCI LTD). (**b**) Schematic diagram of selective etching of Al layer from MAX phase to form Ti_2_CT_x_ MXene [34] (copyright 2015, Springer Nature).

MXenes are a new family of 2D materials with the chemical formula M_n+1_X_n_T_x_, where M stands for the transition metal from the IIIB, IVB, VB, and IVB groups, X stands for carbon or nitrogen, *n* is usually 1, 2, or 3, and T_x_ refers to the terminations on the surface [25,27,28]. MXenes are usually prepared in a two-step process. The first step is the synthesis of the MAX phase, which is accomplished by a high-temperature reaction following the chemical formula M_n+1_AX_n_, where A represents the elements from the IIIA or IVA groups (such as Al or Si) (Figure 1a) [33]. Common methods include hot-press sintering and arc melting. The second step is the selective etching of the MAX phase to remove the A layer (e.g., Al from Ti_2_AlC) and obtain M_n+1_X_n_T_x_ (Figure 1b) [34]. This process produces functional groups (–O, –OH, –F, –S, –Cl, etc.) on the external M-plate of the 2D layered MXenes (Figure 1b). After etching, monolayers or few-layer MXenes are obtained by ultrasonic exfoliation.

Since the discovery of Ti_3_C_2_T_x_-MXene in 2011, the preparation methods and application fields of MXenes have been expanding. Their large specific surface area (250~1000 m^2^ g^−1^), high conductivity (even up to 2.4 × 10^4^ S cm^−1^), and abundant functional terminals make them promising candidates for energy storage applications, such as lithium-ion batteries, sodium-ion batteries, Li-S batteries, and supercapacitors with a broad application prospect [35,36,37]. Applied to Li-S batteries, MXenes can effectively inhibit the shuttle effect by chemisorption with polysulfides by virtue of their rich surface functional groups, which is a thorny issue plaguing the cycling stability of Li-S batteries. MXenes’ high conductivity can improve the electronic conduction of the sulfur electrode, enhancing the utilization of sulfur as an active substance. In addition, sufficient surface termination in MXenes can induce uniform nucleation and inhibit the growth of lithium dendrites [38,39]. Also, due to the good mechanical stability of MXenes, they can accommodate the large volume change of sulfur species during charging and discharging. In short, MXenes combine all of these advantages into one compared with other 2D materials (graphene, layered hydroxides, etc.). Therefore, MXenes are considered to have strong potential for manufacturing high-performance Li-S batteries.

### 2.2. Properties of MXene-Based Heterostructures

MXenes demonstrate unique appeal in Li-S batteries due to their excellent physicochemical properties. However, their practical application is hindered by the inherent self-restacking tendency of layered MXene sheets, which severely compromises the vertical electron/ion transport pathways between the layers, leading to a degraded electrochemical performance under high sulfur loading [40]. Furthermore, a single-component system of MXene struggles to simultaneously optimize the physical confinement, chemical adsorption, and catalytic conversion of LiPSs, resulting in only marginal improvements in the overall performance of a battery. To address these limitations, the construction of MXene-based heterostructures has been proposed as a strategic approach. By combining MXene with complementary functional materials, synergistic effects can be engineered to tailor specific physicochemical properties (hierarchical porosity, interfacial charge redistribution, multiple active sites, etc.), thereby fulfilling the multifaceted requirements of advanced Li-S battery systems [41,42].

Heterostructure is a concept widely used in multidisciplinary fields, with its theoretical prototype traceable to the classic model proposed by W. Shockley in the field of semiconductor physics [43]. The fundamental features of this structure stem from the natural evolution of heterogeneous interfacial regions, i.e., the interfacial regions are characterized by chemical compositional reconfiguration, charge redistribution, and changes in physicochemical properties due to spatial discontinuities in key parameters, such as differences in the energy band arrangement, Fermi energy level shifts, and carrier concentration gradients [44,45]. In the field of advanced material engineering, heterostructure refers to a composite system that integrates two or more materials with different crystal structures through precise interfacial engineering, the core of which lies in the realization of synergistic effects between component materials through controllable interfacial construction, thus breaking through the performance limits of traditional homogeneous materials and achieving a leapfrog enhancement in key properties. As for MXene-based heterostructured hybrid separators, the inherent defects of single-phase MXene can be effectively overcome by the multicomponent synergistic integration strategy. On the one hand, a built-in electric field can be formed by exploiting the energy band engineering of interfacial heterojunctions, which can significantly optimize the ion transport kinetics; on the other hand, the charge redistribution is induced by precisely tuning the interfacial electronic structure to construct a multi-site adsorption–catalytic network for LiPSs, which significantly improves the electrocatalytic performance and the reversible capacity of the Li-S battery system.

## 3. Synthesis of MXenes and MXene-Based Heterostructures

### 3.1. Synthesis of MXenes

In general, the synthesis of MXenes mainly involves two types of strategies: top-down and bottom-up. The top-down method, which is the primary approach for synthesizing MXenes, involves the selective etching of the A layer from MAX precursors, followed by exfoliation into single or multi-layer nanosheets [46]. The commonly used etching methods include HF etching, in situ generation of HF for etching, and molten-salt etching. For the bottom-up method, direct growth of MXene using chemical vapor deposition (CVD) is typical. However, this method is still at an early stage due to strict synthesis conditions and complex processes.

#### 3.1.1. HF Etching

HF was the first etchant used to produce MXenes, and the A layer in MAX dissolves easily in HF. Taking Ti_3_AlC_2_ as an example, the etching reactions are as follows [47]:

Ti_3_AlC_2_ + 3HF = AlF_3_ +Ti_3_C_2_ + 3/2H_2_ (1)

Ti_3_C_2_ + 2H_2_O = Ti_3_C_2_(OH)_2_ + H_2_ (2)

Ti_3_C_2_ + 2HF = Ti_3_C_2_F_2_ + H_2_ (3)

#### 3.1.2. In Situ Generation of HF for Etching

Considering the toxicity of HF, another safer alternative etchant was developed. A mixed solution of LiF/HCl can slowly generate HF, exhibiting the same effect as HF as a mild etchant. Moreover, Li^+^ and H_2_O molecules can be simultaneously inserted into the layered structure during the etching process, which helps to expand the interlayer distance and the subsequent exfoliation of the MXene nanosheets [48].

#### 3.1.3. Molten-Salt Etching

Different from acid solution etching, molten-salt etching at high temperatures does not require the use of HF or fluoride-containing acidic solutions, which improves the safety of the synthetic process and reduces waste liquid treatment costs. In the high-temperature molten-salt etching method, appropriate Lewis acid chloride molten salts, such as ZnCl_2_, CuCl_2_, etc., are typically selected as etchants. For example, a MAX precursor was mixed with CuCl_2_, NaCl, and KCl in specific ratios and then heated for 24 h at 680 °C in an argon atmosphere to remove the A atoms [49].

#### 3.1.4. CVD Method

The bottom-up strategy builds from atoms to a layered structure, and the CVD method adopts this approach to synthesize MXene. For instance, Wang and colleagues [50] synthesized Ti_2_CCl_2_ via CVD. The carpet-like Ti_2_CCl_2_ sheets, perpendicular to the metal, were formed through the reaction of methane and titanium tetrachloride on the titanium surface. These MXene carpets underwent buckling under certain growth conditions, forming vesicles that separated from the substrate.

### 3.2. Synthesis of MXene-Based Heterostructures

#### 3.2.1. Hydrothermal/Solvothermal Reaction

The hydrothermal/solvothermal method is the in situ growth of oxides, sulfides, or selenides, etc., on the surface of MXene by promoting precursor decomposition and crystallization using solvents in a closed high-temperature and high-pressure environment. For example, a NiS_2_/Ti_3_C_2_T_x_ heterostructure was synthesized through a two-step hydrothermal route. Ni ions were in situ self-assembled on the surface of Ti_3_C_2_T_x_ nanosheets during the first hydrothermal process, facilitated by the abundant surface groups (including –OH, –O, etc.). Subsequently, NiS_2_/Ti_3_C_2_T_x_ was obtained through a hydrothermal sulfuration reaction [51].

#### 3.2.2. Electrostatic Self-Assembly

Due to the presence of negatively charged functional groups, such as –OH, –O, and –F, on the surface of MXene nanosheets, they can easily adsorb positively charged ions or materials through electrostatic interaction. Subsequently, MXene-based heterostructures can be formed either through in situ self-assembly or chemical reaction on the MXene surface. Post-treatment operations, such as carbonization or phosphorization, can also be combined for further transformation. For example, positively charged Co and Ni ions were adsorbed onto the negatively charged surfaces of MXenes through electrostatic interactions. Then, assisted by NH_3_ H_2_O, the NiCo-precursor underwent in situ self-assembly on the MXenes’ surfaces to form an LDHs-MXenes heterostructure [52].

#### 3.2.3. Lewis Acid Etching and Subsequent Chemical Conversion

Through Lewis acid molten-salt etching, the A-site in the MAX phase can be replaced by transition metal atoms, introducing tightly anchored metal particles on MXenes, which are then transformed into heterostructures through chemical conversions such as selenization or sulfurization. For example, Ti_3_AlC_2_ and CoCl_2_ were reacted to synthesize a Ti_3_C_2_T_x_@Co hybrid at 750 °C in a molten state. Due to the coordinatively unsaturated nature, Co^2+^ is a strong electron acceptor and serves as a Lewis acid in molten salt, providing an acidic environment and breaking the weak bonds of Al atoms in Ti_3_AlC_2_. Meanwhile, Co^2+^ was reduced in situ. The Ti_3_C_2_T_x_@CoSe_2_ heterostructure was then obtained through a selenization process [53].

#### 3.2.4. Partial Transformation of MXenes

The partial transformation of MXene is realized by means of oxidation, etc., to form a heterostructure. For example, the oxidation treatment of Ta_4_C_3_ MXene using dilute H_2_O_2_ resulted in the significant exposure of Ta atoms on its surface. Subsequent calcination under an Ar/H_2_ atmosphere resulted in the formation of a Ta_4_C_3_-Ta_2_O_5_ heterostructure [54].

#### 3.2.5. Vacuum Filtration Self-Assembly

The dispersion of MXene with other nanomaterials was vacuum-filtered into a film to form a layered heterostructure. For example, a uniform dispersion of MXene/deoxyribonucleic acid (DNA)-CNT was obtained through a blending process. In the subsequent vacuum filtration process, driven by self-assembly, interconnected networks were formed between DNA and MXene through π–π stacking and coordination interactions, leading to the formation of a DNA-CNT/MXene heterostructure [55].

## 4. Construction of MXene-Based Heterostructured Hybrid Separators

MXene-based heterostructures can prevent the self-stacking defects of MXene nanosheets to fabricate three-dimensional (3D)/2D conductive heterostructures. The built-in electric field formed at the heterogeneous interfaces can accelerate the migration of charge carriers, the diffusion of lithium ions, and the anchoring/catalysis of polysulfides. Given the excellent properties of MXene and the superior multifunctional components, this structure serves as a functional material to modify PP separators, enabling outstanding Li-S battery performance. This section will summarize the recent research progress on MXene-based heterostructured hybrid separators in terms of MXene/inorganic metal, MXene/inorganic nonmetal, MXene/organic framework and polymer, and MXene/carbon heterostructured materials.

### 4.1. MXene/Inorganic Metal Heterostructures

Polar-metal materials, including metal sulfides, selenides, oxides, and phosphides, show great potential in capturing polysulfides and catalytically accelerating their conversion reactions to improve redox kinetics. However, separators modified with metal compounds alone exhibit an inadequate electrochemical performance due to their relatively poor conductivity, their single chemical adsorption of LiPSs, and the tendency of nanoparticles to aggregate. Combining metal compounds with high-conductivity MXene substrates to construct heterostructured hybrid separators offers significant advantages.

#### 4.1.1. MXene/Metal Sulfide Heterostructures

**Figure 2:**
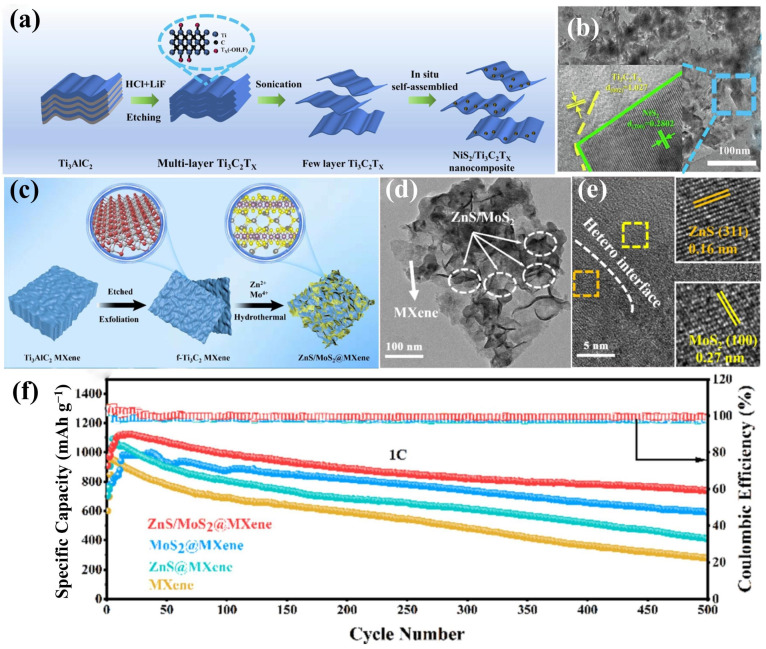
(**a**) Diagrammatic representation of the synthetic procedure; (**b**) TEM and HRTEM (inset) images of the NiS_2_/MXene heterostructure [51] (copyright 2024, Elsevier). (**c**) Diagrammatic representation of the synthetic procedure, (**d**) TEM and (**e**) HRTEM images of the ZnS/MoS_2_@MXene heterostructure, and (**f**) long-term cycling test at 1C of the ZnS/MoS_2_@MXene hybrid separator [63] (copyright 2024, Elsevier).

Metal sulfides, especially transition-metal sulfides, are highly effective in regulating the shuttle effect due to their strong chemical adsorption of LiPSs. This can be attributed to the special polarized surface generated by the metal-S polar bonds, as well as the strong chemical interaction between the metal sulfides and LiPSs [51]. They also serve as efficient catalysts in Li-S batteries, particularly when combined with MXene, and are highly favored. Wang et al. [51] synthesized a NiS_2_/MXene (Ti_3_C_2_T_x_) heterostructure with mesoporous structures through the two-step hydrothermal reaction (Figure 2a), in which 0D ultrafine NiS_2_ nanoparticles were in situ self-assembled onto 2D conductive MXene nanosheets (Figure 2b). Thanks to the enhanced chemical adsorption, abundant catalytic active sites, and high electronic conductivity of the NiS_2_/MXene-modified separator, the hybrid separator exhibited remarkable characteristics in capturing and converting LiPSs and achieved efficient transfer pathways for electrons/ions. As a result, Li-S batteries with modified separators demonstrate an outstanding rate performance and cycling stability. Transition-metal disulfides, such as CoS_2_ [56], MoS_2_ [57], WS_2_ [58], and VS_2_ [59], as well as VS_4_ [60], ZnS [61], and Co_1-x_S [62], when combined with MXene to form heterostructured hybrid separators, have also demonstrated good performance in Li-S batteries. Single-component active materials usually struggle to balance the “adsorption–catalysis” throughout the entire redox process due to the multi-stage phase transitions of sulfur. Accordingly, multi-heterostructured catalysts have been emerging, and their multi-component synergistic catalytic effect on the adsorption–conversion of LiPSs is remarkable. Li et al. [63] prepared the heterostructure of ZnS/MoS_2_@MXene nanosheets uniformly anchored on MXene by hydrothermal and ultrasonic processes (Figure 2c–e). The heterogeneous interface of ZnS/MoS_2_@MXene can promote the efficient adsorption, catalysis, and conversion of LiPSs, thereby suppressing the shuttle effect and significantly enhancing the sulfur redox kinetics. The Li-S battery with the ZnS/MoS_2_@MXene modified separator achieved 1504.4 mAh g^−1^ at 0.1 C and still maintained a high discharge capacity of 741 mAh g^−1^ after 500 cycles at 1C (Figure 2f). There is also the Bi_2_S_3_/MoS_2_@MX heterostructure studied by Li et al. [31], the MXene/MoS_2_/SnS@C heterostructure studied by Cui et al. [64], and MXene/NiS_2_/Co_3_S_4_ heterostructure reported by Wang et al. [65]. In the research by Li et al. [31], attributed to the synergistic effect of the heterostructure, the sulfur utilization was enhanced, and the corrosion of the lithium anode was reduced. Ultimately, the battery demonstrated an excellent long-term cycling performance, with a decay rate of only 0.069% per cycle for 500 cycles at 2 C.

#### 4.1.2. MXene/Metal Selenide Heterostructures

**Figure 3:**
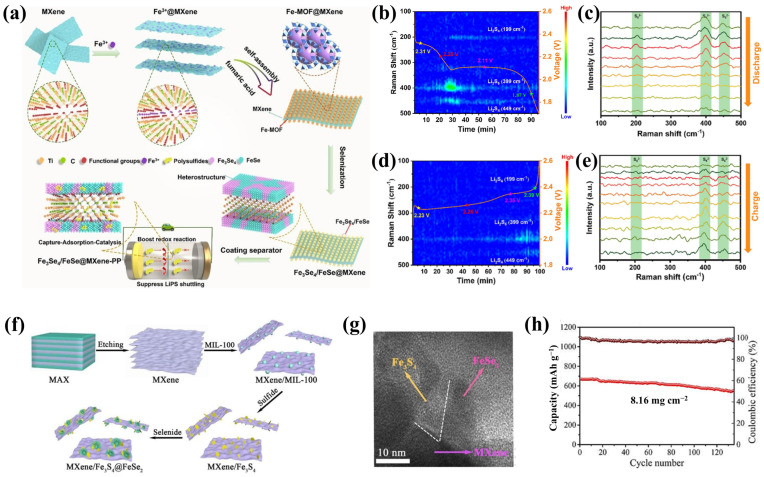
(**a**) Depiction of the Fe_3_Se_4_/FeSe@MXene synthetic procedure; (**b**–**e**) in situ Raman spectra and Raman contour plots at 0.5C for Fe_3_Se_4_/FeSe@MXene-PP [69] (copyright 2023, Elsevier). (**f**) Synthetic procedure, (**g**) HRTEM image of MXene/Fe_3_S_4_@FeSe_2_, and (**h**) high sulfur loading performance of Li-S battery combined with MXene/Fe_3_S_4_@FeSe_2_ [70] (copyright 2023, Elsevier).

Transition-metal selenides not only possess high electrical conductivity and strong bonding with LiPSs, but their unique d-electron configuration also endows them with exceptional electrocatalytic properties for LiPS conversion [66]. Research has shown that CoSe_2_ possesses conductivity comparable to metals and can effectively capture LiPSs through strong S-Co and Li-Se bonds [67,68]. For example, Han’s team [53] prepared a heterostructure with CoSe_2_ nanoparticles strongly anchored on an MXene substrate through Lewis acidic etching and subsequent selenization. The Ti_3_C_2_Tx@CoSe_2_ heterostructure exhibited high conductivity and catalyzed the conversion of LiPSs. The heterostructured hybrid separator significantly improved the performance of Li-S batteries. In addition, Zhou et al. [69] synthesized an Fe_3_Se_4_/FeSe@MXene heterostructure by in situ growth and selenization (Figure 3a). The MXene framework effectively captured polysulfides, while the Fe_3_Se_4_/FeSe heterostructure primarily served a catalytic role. Density functional theory (DFT) calculations confirmed that the interface engineering of Fe_3_Se_4_/FeSe increased the density of states at the Fermi level and reduced the activation energy for Li_2_S decomposition, and the internal electric field accelerated the charge transfer. In situ Raman spectroscopy detected the changes in the intensity of S_6_^2−^ during the discharge/charge process, confirming the good reversibility of the electrochemical reaction (Figure 3b–e).

Differing from the above, Zhang’s group [70] designed multi-heterostructured MXene/Fe_3_S_4_@FeSe_2_ catalysts using a combination of vulcanization and selenization, which consisted of single-layer MXene, Fe_3_S_4_ nanosheets, and FeSe_2_ nanoclusters (Figure 3f). The results confirmed that the heterostructure between MXene and Fe_3_S_4_ was constructed through chemical bonds, forming free charge carriers on both sides of the contact interface, resulting in an internal electric field. A large number of unsaturated sites contributed to the formation of a vacancy-rich heterostructure of Fe_3_S_4_ and FeSe_2_ (Figure 3g). As such, this heterostructure was rich in multiple catalytic active centers, exhibiting a good synergy of conduction–adsorption–catalysis. The Li-S battery assembled with MXene/Fe_3_S_4_@FeSe_2_ achieved an initial discharge capacity of 1185.6 mAh g^−1^ at 0.2 C. Under a high sulfur loading of 8.16 mg cm^−2^, the capacity retention after 135 cycles was as high as 82.4% (Figure 3h).

#### 4.1.3. MXene/Other Inorganic Metal Heterostructures

**Figure 4:**
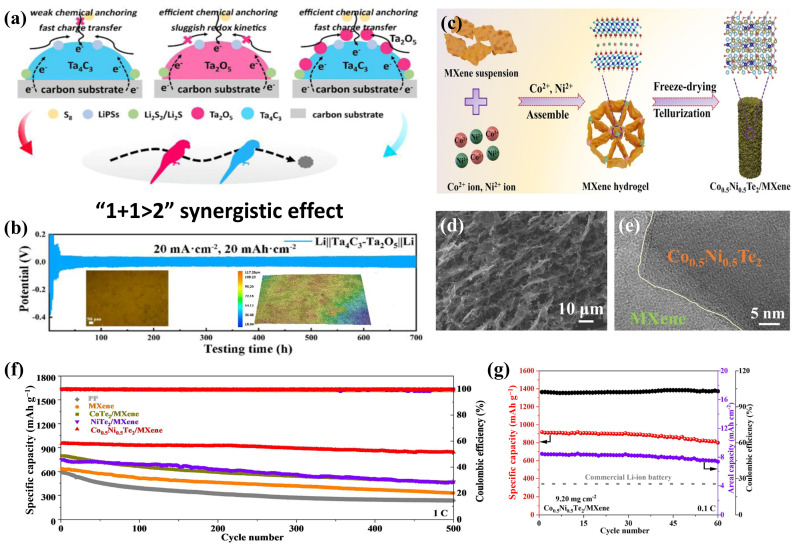
(**a**) Structural characteristics of the components between Ta_4_C_3_ and Ta_2_O_5_; (**b**) voltage–time profiles of symmetrical cells at 20 mA cm^−2^ for 20 mAh cm^−2^ (inset: corresponding 3D optical profilometry images of Ta_4_C_3_-Ta_2_O_5_@C-PP on Li metal surface after cycling [54]) (copyright 2023, Chinese Society of Metals). (**c**) Synthetic procedure, (**d**) SEM and (**e**) HRTEM images of Co_0.5_Ni_0.5_Te_2_/MXene heterostructure, and (**f**) long cycling performance at 1 C and (**g**) high sulfur loading test at 9.20 mg cm^−2^ [74] (copyright 2024, Elsevier).

Metal oxides possess high polar surfaces due to metal–oxygen (M-O) bonds, which can chemically bond with LiPSs, thereby fixing sulfur and inhibiting the shuttle effect [71]. Lee et al. [72] successfully designed a nano-whisker-like H_2_Ti_3_O_7_ grown on MXene (M-HTO-0.5) using MXene-derived hydrogenation and ion exchange processes. The multifunctional synergistic effect of the M-HTO-0.5 heterostructured hybrid separator effectively limited the shuttle of LiPSs and the growth of lithium dendrites. As a result, the Li-S battery with the hybrid separator could achieve a stable long-term cycling performance even at 5.0 C. Wang et al. [73] improved the surface structure and electronic distribution of MXene by introducing Nb atoms. They synthesized a Ti_3−y_Nb_y_C_2_T_x_/TiO_2_ heterostructure (O-Ti_3−y_Nb_y_C_2_T_x_) by in situ oxidizing Ti_3−y_Nb_y_C_2_T_x_ MXene in anhydrous ethanol, which was applied in the separators and cathodes of Li-S batteries, demonstrating a superior performance. In addition, a one-stone–two-birds strategy to suppress the shuttling of polysulfides and protect the lithium anode was proposed by Liang et al. [54], which involved constructing a Ta_4_C_3_-Ta_2_O_5_ heterostructured hybrid separator. As shown in Figure 4a, this novel MXene (Ta_4_C_3_) derivative heterostructure effectively combined the structural advantages of Ta_4_C_3_ and Ta_2_O_5_, providing a synergistic effect for LiPS capture and redox reactions. Furthermore, even at a high current density of 20 mA cm^−2^/20 mAh cm^−2^, the hysteresis voltage of the Li||Ta_4_C_3_-Ta_2_O_5_||Li symmetric cell could still maintain a stable plateau curve (Figure 4b). The optical profilometry images (insets in Figure 4b) also indicate that the lithium surface of Ta_4_C_3_-Ta_2_O_5_@C-PP exhibits uniformity and low roughness after plating. Metal tellurides have attracted special research interest due to their unique ionic transport dynamics and high conductivity. Li et al. [74] constructed a catalytic 3D Co_0.5_Ni_0.5_Te_2_/MXene heterostructure through a gelation process assisted by bimetallic ions (Ni^2+^ and Co^2+^) and subsequent tellurization (Figure 4c–e). Notably, Ni^2+^ and Co^2+^ served as cross-linking agents, collaboratively achieving and regulating the 3D macroporous structures (Figure 4d), thereby enriching the multifunctional active sites. As a result, the Li-S battery with a Co_0.5_Ni_0.5_Te_2_/MXene hybrid separator showed a minimum decay rate of only 0.0227% per cycle for 500 cycles at 1 C (Figure 4f). Even under high sulfur loading (9.20 mg cm^−2^) and low electrolyte/sulfur ratio (E/S = 6.2 µL mg^−1^) (Figure 4g), the battery could achieve a capacity of 8.46 mAh cm^−2^, which clearly exceeds the capacity of typical commercial lithium-ion batteries (areal capacity of 4 mAh cm^−2^) throughout the entire cycle. Furthermore, there are also heterostructured hybrid separators constructed with MXene, such as Co_x_P [75,76], Co_2_B [77], WSSe [78], and layered double hydroxides (LDHs) [52], all of which can achieve comparable effects. Additionally, some MXene-derived metallic compound heterostructures, such as Co@M-TiO_2_/C [79], HPCA-TO [80], TiN@C [81], and CoFe/FeN_0.0324_/TiN [82], could be easily constructed and were effective for trapping LiPSs and accelerating redox kinetics when used as functional separator coatings.

The MXene/inorganic metal heterostructured hybrid separators for Li-S batteries are summarized in Table 2. It can be seen that studies on MXene/metal sulfide heterostructures account for a significant portion of the field. This can be attributed to the strong anchoring–catalytic ability of sulfides toward LiPSs, as well as the excellent structural compatibility between MXene and sulfides, which facilitates the formation of tight 2D/2D heterointerfaces. Additionally, the synthetic process of sulfides (e.g., hydrothermal) is highly compatible with the liquid-phase processing of MXene. In general, the unique polar surface of inorganic metallic materials can significantly inhibit shuttling through strong chemical adsorption, resulting in Li-S batteries with MXene/inorganic metal heterostructured hybrid separators exhibiting an initial capacity generally above 900 mAh g^−1^ at a relatively high rate of 1C. The superiority of such materials lies in the enhanced catalytic conversion kinetics due to the interfacial electronic coupling of metal ions with different valence states. However, the heterostructures of MXene with other inorganic metals remain to be explored, and the volume expansion leading to limited long-term cycling stability has to be considered for the metal components.

### 4.2. MXene/Inorganic Non-Metal Heterostructures

**Figure 5:**
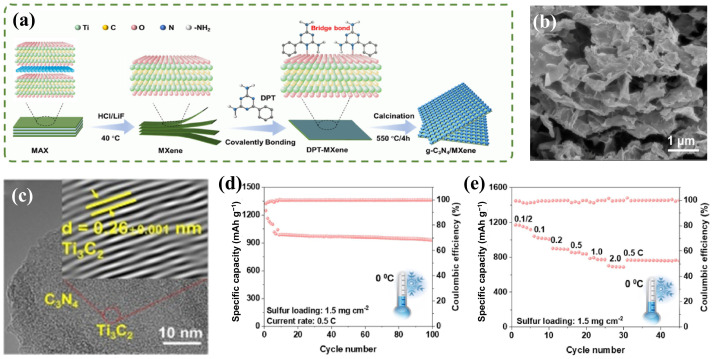
(**a**) Diagrammatic representation of creation, (**b**) SEM and (**c**) HRTEM images of g-C_3_N_4_/MXene heterostructure, (**d**) cycling test, and (**e**) rate performance of batteries with CN-MX in a cold 0 °C setting [83] (copyright 2024, Academic Press Inc.).

g-C_3_N_4_ is a unique 2D carbon nitride compound with abundant intrinsic defects and highly polarized sites commonly used in Li-S batteries [83]. However, the excessive substitution of nitrogen atoms in the carbon network significantly weakens the conductivity of g-C_3_N_4_. In light of this, Wang et al. [83] synthesized a 2D porous g-C_3_N_4_/MXene (CN-MX) heterostructure with numerous intrinsic defects through electrostatic adhesion and in situ thermal polycondensation (Figure 5a–c). MXene nanosheets act as charge transport channels, while the CN layer ensures the rapid dissociation of solvated Li^+^ and the smooth diffusion of free Li^+^. The heterogeneous interface also provides active sites to accelerate redox reactions. The CN-MX heterostructure-modified separator exhibited excellent Li-S battery performances, especially under low-temperature and high-sulfur-loading conditions. At a low temperature of 0 °C, the capacity retention rate of the cell was as high as 91.6% at 0.5 C and reached 695 mAh g^−1^ at 2.0 C (Figure 5d,e). This work provides new ideas for MXene-based heterostructured hybrid separators in Li-S batteries to adapt to various extreme operating conditions and realize energy storage batteries with excellent performances and low cost. BN is a low-bandgap semiconductor material with strong adsorption and catalytic capabilities, and the heterogeneous structures of BN and MXene are potentially complementary [84]. Wang’s team [84] designed a BN@Mxene heterostructure with a staggered layered structure as a functional interlayer of the separator. This unique structure provides a pathway for the smooth transportation of Li^+^ and physically blocks the shuttle of LiPSs to some extent. In addition, the effective catalytic role of BN@MXene in the conversion of Li_2_S_6_ to Li_2_S was demonstrated by studying the change in Gibbs free energy and the optimized absorption models during the reduction of S_8_ on BN@MXene and MXene. The Li-S battery with a BN@MXene heterostructured interlayer delivered a reversible charge capacity of 404.9 mAh g^−1^ after 700 cycles at a current density of 1 C, with an extremely low decay of 0.058% per cycle.

Inorganic non-metals, represented by 2D g-C_3_N_4_ and BN (Table 2), are suitable complementary heterostructures with MXene due to their peculiar inherent defects and polar surface groups. Yet, most of the inorganic non-metals have low intrinsic conductivity and weak adsorption ability for LiPSs, which leads to insufficient capability in interfacial charge transfer and polysulfide anchoring when forming heterostructures with MXene. Given this, studies on MXene/inorganic non-metal heterostructured hybrid separators are limited, but their special merits in thermal stability and mechanical strength still make them promising, requiring further development.

### 4.3. MXene/Organic Framework and Polymer Heterostructures

**Figure 6:**
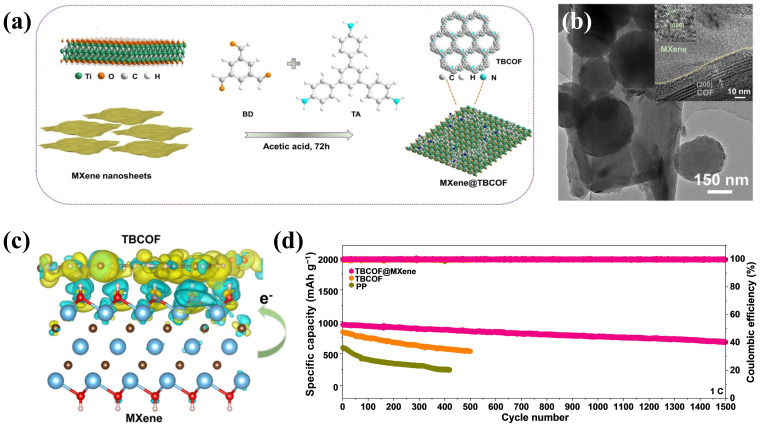
(**a**) Synthetic procedure, (**b**) TEM image (inset: HRTEM image) of MXene@TBCOF, (**c**) differential charge density diagram of the interaction between TBCOF and MXene, and (**d**) long cycling performance at 1 C [32] (copyright 2024, Elsevier).

Two-dimensional covalent organic frameworks (COFs) represent one type of porous material characterized by robust covalent bonds and periodic structural units [85,86], featuring high porosity, excellent stability, and tunable chemical properties [87,88]. The well-defined porous configuration of COFs facilitates the diffusion of lithium ions directly, while N atoms function as lithium-affinitive sites, improving the interface contact with LiPSs and thereby offering significant potential for Li-S batteries [32]. Regrettably, the electronic insulating properties and low redox behavior of COFs significantly hinder the swift transformation of LiPSs. The creation of an MXene/COF heterostructure, an organic–inorganic hybrid material, merges a superior pore architecture with elevated conductivity, forming a complementary interface on the separator of Li-S batteries. Yang et al. [89] enabled the slow growth of COFs with two different pore sizes inside and on the surface of MXene nanosheets by an in situ polymerization reaction. The N sites in the COF monomer were covalently bonded to the Ti sites exposed on the surface of MXene, thus realizing the bridging. This promoted the complementary strengths of MXene and COF to facilitate rapid polysulfide conversion. The suitable pore structure could limit polysulfide shuttling while regulating Li^+^ flux for uniform lithium deposition. As expected, the MXene@COF integrated separator enabled Li||Li symmetric batteries to achieve up to 4750 h of stable lithium plating/stripping at 10 mA cm^−2^. Also, the assembled Li-S batteries had a high capacity of 584/563 mAh g^−1^ at 3 C. Moreover, in the work by Li et al. [32], TBCOFs were obtained by the Schiff-base condensation reaction and then in situ grafted onto the MXene surface at room temperature. As shown in Figure 6a, spherical TBCOF particles grew uniformly on the surface of the MXene nanosheets and formed the MXene@TBCOF heterostructure (Figure 6b). This symbiotic partnership between MXene and TBCOF diminished the Li-S bonds, thereby concurrently suppressing the shuttle effect of LiPSs and acting as a precision sieve for the regulation of ion transport. DFT calculation is displayed in Figure 6c. The blue isosurfaces represent the decrease of the charge density and the yellow regions denote the accumulation of electron density. It is clear that at the TBCOF@MXene heterogeneous interface, more electrons accumulate on the TBCOF side, while the MXene side retains a large number of electron holes (positive charges), suggesting that the presence of a gradient built-in electric field within MXene@TBCOF, which enhances the efficiency of lithium-ion transportation. A battery equipped with an MXene@TBCOF separator achieved excellent cyclability at a current density of 1C over 1500 cycles with a capacity decay rate of only 0.0191% per cycle (Figure 6d). Even with a sulfur content as high as 9.31 mg cm^−2^, the battery showed an exceptional area capacity of 7.14 mAh cm^−2^ after 90 cycles. Similarly, Li’s group [90] constructed a PP separator coating consisting of guanidinium-based ionic–covalent organic nanosheets (iCON) uniformly loaded on Ti_3_C_2_ nanosheets (Ti_3_C_2_@iCON) to exert electrostatic attraction and catalytic effects on polysulfides. The modified separator was effective even at a sulfur content of 90 wt% and a sulfur loading of 7.6 mg cm^−2^. Additionally, based on the excellent porosity and abundant active sites of the metal–organic frameworks (MOFs), their combination with the high conductivity of MXene can synergistically enhance the performance of Li-S batteries [91,92]. Maryam Sadat Kiai’s research team [93] adopted this idea by coating a glass fiber separator with a conductive Ti_3_C_2_T_x_ nanosheet/Fe-MOF or Ti_3_C_2_T_x_ nanosheet/Cu-MOF layer as the polysulfide trapping layer. Short- and long-chain polysulfides captured on the surface of the separator were reduced to sulfur by accepting electrons from the MXene to improve sulfur utilization. Porous Fe/MOF and Cu/MOF could be used as lithium-ion sieves to intercept polysulfides and reduce the growth of lithium dendrites. The Li-S batteries achieved significant improvements in discharge capacity, cycle stability, and electrochemical conversion through this clever design.

**Figure 7:**
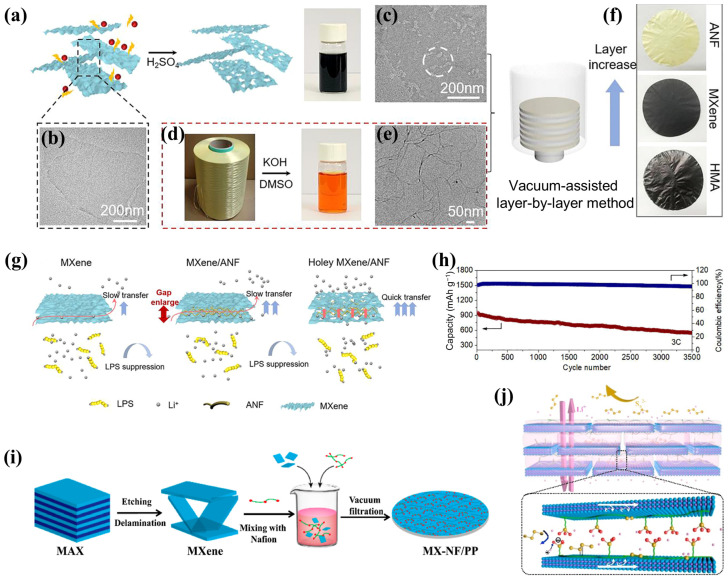
(**a**) Synthetic procedure of porous MXene nanosheet, TEM images of (**b**) MXene and (**c**) holey MXene nanosheets, (**d**) crafting the ANF using commercial Kevlar yarns, (**e**) TEM image of ANF, (**f**) photograph of ANF, MXene, and HMA membrane, (**g**) diagram of ion transfer in different separator, and (**h**) long cycling performance at 3 C [94] (copyright 2023, Elsevier). (**i**) Preparation method and (**j**) diagrammatic depiction of the roles of MX-NF/PP separator [95] (copyright 2019, Elsevier Ltd.).

Alternatively, multilayer coatings of MXene/organic polymer material separators can be prepared by the vacuum-assisted layer-by-layer assembly process. Wang et al. [94] formed a stable suspension of porous MXene obtained by etching (Figure 7a–c) and aramid nanofiber (ANF) (Figure 7d,e), followed by layer-by-layer deposition by vacuum filtration to prepare the multilayer holey MXene/ANF (HMA) separator (Figure 7f). The etching process produced hierarchical nanoporous structures (15–30 nm) and various functional groups (–OH; –O) on the surface of the MXene substrate, which shortened the ion channels in the re-stacked films and enhanced the adsorption of LiPSs. The negative charges of the ANF widened the interlayer spacing between the porous MXene nanosheets and prevented them from re-stacking, which further facilitated the ion transport, as illustrated in Figure 7g. The Li-S batteries with the HMA separator had a long cycle life of 3500+ cycles at 3 C with a capacity decay of 0.013% per cycle (Figure 7h). By a similar approach, Wang’s group [95] deposited MXene and Nafion chains onto one side of a PP separator (Figure 7i), attributing that the physical resistance of MXene and the cationic permselectivity of Nafion could synergistically inhibit the shuttling effect to achieve the good cycling stability of the Li-S batteries (Figure 7j). Specifically, the −SO_3_^−^ groups on Nafion produced strong electrostatic repulsion for negatively charged polysulfides and simultaneously electrostatic attraction for positively charged Li^+^, so Nafion has cation permselective effect that ensured rapid Li^+^ transport and inhibited polysulfide transmission. The work highlighted the full synergistic effect of organic and inorganic components in such layered nanostructures. Furthermore, taking the layered GO-Nafion as an example, the research further validated the versatility of this mindset. Furthermore, the monocarboxylic pillar [5] arene (PA5-COOH) was successfully grafted onto Nb_2_CT_x_ through an esterification reaction, resulting in the formation of the PA5-COOH/Nb_2_C hybrid separator. This design expanded the application potential of organic macrocyclic molecules [96].

The electrochemical performances of MXene/organic framework and polymer heterostructured hybrid separators are organized in Table 3. Such structures realize multi-level channels through the controllable modification of organic functional groups, effectively improving ionic conductivity while maintaining excellent flexibility. However, the low conductivity of the organic components necessitates ensuring a relatively thin coating (with a loading of less than 0.3 mg cm^−2^), and electrochemical instability hinders their long-term cycling performance.

### 4.4. MXene/Carbon Heterostructures

Carbonaceous materials offer the advantages of light weight, excellent electrical conductivity, large specific surface areas, and superior electrochemical stability to immobilize sulfur species, mitigate their volume change, and enhance electrochemical kinetics [97,98]. Carbonaceous materials such as carbon nanotubes (CNTs), graphene oxide (GO), and 3D porous carbon are introduced into MXene, which not only form crosslinked electron pathways but also act as spacer insertion agents to prevent the buildup of MXene nanosheets [99,100,101]. As such, MXene/carbon material heterostructures have excellent performance in Li-S battery applications, which can effectively inhibit the shuttle effect of polysulfides and improve the cycling stability and capacity of cells.

**Figure 8:**
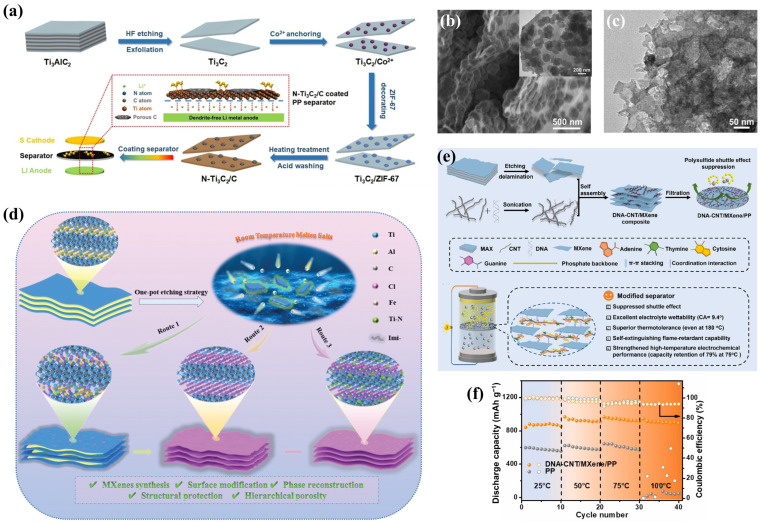
(**a**) Diagrammatic representation of the creation of N-Ti_3_C_2_/C and the modified separator, (**b**) SEM image (inset: TEM image) of Ti_3_C_2_/ZIF-67, and (**c**) TEM image of N-Ti_3_C_2_/C [102] (copyright 2019, Elsevier). (**d**) Diagrammatic representation of etching routes for intermediary products of Ti_3_AlC_2_ MAX, fluorine-free Ti_3_C_2_T_x_, and fluorine-free Ti_3_C_2_T_x_ featuring Ti in coordination with N [104] (copyright 2023, Wiley-VCH). (**e**) Diagram of synthetic strategy and action mechanism of DNA-CNT/MXene/PP separator and (**f**) electrochemical stability of PP and DNA-CNT/MXene/PP cells at different temperatures [55] (copyright 2023, Elsevier Ltd.).

Jiang et al. [102] prepared a nitrogen-doped Ti_3_C_2_/carbon 2D heterostructure (N-Ti_3_C_2_/C) as a commercial PP separator coating by in situ decorating ZIF-67 nanoparticles on 2D ultra-thin Ti_3_C_2_ nanosheets and subsequent heat treatment (Figure 8a). It should be emphasized that, as shown in Figure 8b, the surface of Ti_3_C_2_ was not fully covered by the ZIF-67 nanoparticles but left a large active Lewis acid adsorption surface. The surfaces of the prepared N-Ti_3_C_2_/C 2D nanosheets showed a highly developed porous structure (Figure 8c). N-Ti_3_C_2_/C@PP could not only effectively prevent the shuttling of polysulfides but also successfully inhibited the growth of lithium dendrites, enabling Li-S batteries to exhibit good electrochemical performances. Shao’s research team [103] prepared a carbon-coated nitrogen and vanadium co-doped MXene (denoted as CNVM) separator coating using a simple one-pot mixing and calcination method. The structure of MXene changed due to the doping of heteroatoms, creating more anchoring sites for LiPSs and promoting the migration of charge carriers. The conductivity was further enhanced by the N-doped carbon while also increasing its chemical affinity with LiPSs. The pouch cells assembled with CNVM could still maintain a stable electrochemical performance and excellent operational characteristics under the high sulfur loading of 7.2 mg cm^−2^, which demonstrated its practical potential. It is worth mentioning that Zhang and colleagues [104] adopted an innovative one-pot etching method to successfully prepare a fluorine-free Ti_3_C_2_T_x_ with hierarchical porous nitrogen-doped carbon (HPNC) encapsulation, which exhibited adjustable coordination chemistry characteristics. Figure 8d shows a schematic of a gradually etching Ti_3_AlC_2_ MAX using room-temperature molten salt (RTMS), which includes the intermediate products of etching (route 1), fluorine-free Ti_3_C_2_T_x_ (route 2), and fluorine-free Ti_3_C_2_T_x_ equipped with the Ti-N structural coordination (route 3). The unique coordination structure between Ti and N in Ti_3_C_2_ MXene arises from Ti-based phase reconstruction. The synergistic effect between the Ti-N coordination structure and HPNC enhances the binding energy, reduces the energy barrier, accelerates the redox kinetics, and helps in the stable fixation of polysulfides. This work provides an innovative and universal etching method for synthesizing fluoride-free MXenes with a tunable coordination chemistry.

There are also MXene/carbon hybrid separators produced using vacuum filtration technology, in which the MXene and carbon materials are pushed and stacked or combined in certain ways to form structures with heterogeneous properties through meticulous design and operation. Li et al. [55] first modified CNTs using DNA, which resulted in the uniform distribution of CNTs in water due to the formation of intermolecular π–π stacking between the aromatic bases in the DNA and the unpaired electrons on the surface of the CNTs under ultrasound. Then, the DNA-CNT/MXene/PP hybrid separator was obtained by the vacuum filtration of the aqueous solution of MXene and DNA-CNT, as shown in Figure 8e. There are multiple interactions between DNA molecules and MXene nanosheets. More specifically, the base pairs/phosphate backbone of DNA interact with the graphite structure/titanium elements of MXene through π–π stacking and coordination, forming an interconnected network (see Figure 8e). It is for this reason that the DNA-wrapped CNTs are anchored to the surface of the MXene nanosheets, which not only ensures the smooth flow of the conductive channels but also prevents the re-stacking of the MXene nanosheets. This unique structure not only has an excellent effect on the shuttle of polysulfide and electrolyte wettability but also exhibits high thermal resistance (even at 180 °C) and flame retardancy. This results in the excellent operating performance of Li-S batteries applying the modified separator over a wide temperature range from 25 to 100 °C (Figure 8f). Similarly, CNTs/MXene-PP [105] and Ti_3_C_2_T_x_/CNTs 10%-PP [106] hybrid separators were also fabricated by CNTs’ surface modification combined with vacuum filtration.

Table 4 encapsulates the electrochemical performance of the MXene/carbon heterostructured hybrid separators. The heterostructure of MXene and carbon material demonstrated a better overall performance. The 3D interconnection of the conductive network enhances the discharge capacity, while the physical–chemical synergistic adsorption effectively reduces the capacity decay rate. The main limitation of this heterostructure lies in the complex preparation process, often requiring high-temperature annealing, which results in high costs.

## 5. Conclusions and Prospects

### 5.1. Conclusions

In summary, we have developed a comprehensive review of the construction of MXene-based heterostructures for the modification of Li-S battery separators, including the categories of MXene/metal sulfides, MXene/metal selenides, MXene/other inorganic metals, MXene/inorganic non-metals, MXene/organic frameworks and polymers, and MXene/carbon materials, and discussed the efficacy of these heterostructured hybrid separators in facilitating polysulfide conversion and enhancing the safety of lithium anodes. MXenes, with their unique surface functional groups and high conductivity, are responsible for anchoring LiPSs and promoting electron conduction, while the inorganic/organic heterogeneous materials on their surface act as excellent catalysts, boosting and regulating the conversion of sulfur and lithium. In addition, the built-in electric field formed at the heterojunction interface can induce charge redistribution, which significantly optimizes ion transport while building a multi-site adsorption–catalytic network for LiPSs. Integrating the aforementioned properties, the Li-S batteries with MXene-based heterostructured hybrid separators are like a tiger with wings, exhibiting an extraordinary electrochemical performance.

For the construction of MXene-based heterostructures for separator modification, firstly, we summarized the general principles for adjusting the surface chemistry of MXenes to enhance their electrochemical performance. MXenes hold a unique allure owing to their tunable transition metals and surface functional groups. The structure and composition of MXenes can be controlled by atomic substitution and solid solution strategies. The incorporation of heteroatoms can induce electron redistribution and refine the surface structure of MXenes, which is conducive to promoting electron transfer at the reaction interface and further improving the electronic conductivity of MXenes. Importantly, the heteroatoms have an ameliorating effect on the catalysis of LiPSs, thus enhancing the electrochemical cycling stability. The functionalization and modification of the terminal groups of MXenes are also effective means to further enhance the catalytic-adsorption effect. Moreover, the tunable coordination chemical environment of MXenes can be gradually realized through an etching process to improve the binding energy and lower the energy barrier, which benefits the stabilization and conversion of polysulfides. Secondly, effective strategies for designing heterostructures were outlined. For the construction of heterostructures, commonly used designs include the in situ growth of heterogeneous materials on the MXene substrate by chemical reaction or electrostatic self-assembly, obtaining heterostructures by Lewis acid molten-salt etching combined with subsequent chemical transformation, partially converting MXene in situ into its derivatives, laying organic precursors around the MXene and then carbonizing to obtain carbon-based heterostructures, etc. Through the above methods, we can reasonably arrange the interface structure, ensuring the synergistic effect between heterogeneous materials and thereby achieving a significant improvement in key performances.

### 5.2. Prospects

A vast accumulation of research has confirmed the enormous potential of MXene-based heterostructure-modified separators in the development of high-performance Li-S batteries. However, the research on MXene-based heterostructures is still progressing steadily in the face of ongoing challenges, while the development of Li-S batteries based on MXene-based heterostructures still requires great effort.

(1) An atomic-scale understanding of MXene termination effects is needed. Although the terminal groups are known to influence MXene’s adsorption and catalytic properties, their site-specific interactions with polysulfides (e.g., their preferential adsorption on –O vs. –F sites) remain ambiguous due to the lack of dynamic operando characterization tools. By integrating operando testing techniques with DFT simulations, it is possible to correlate the real-time distribution of S species with termination-dependent adsorption energies.
(2) A rational design of the heterointerface charge distribution should be developed. The interfacial electric field in MXene-based heterostructures mainly relies on empirical regulation, lacking precise control over the direction and intensity of charge transfer. By designing the Janus-type heterostructure with asymmetric surface terminations, the internal spontaneous electric field can be created to directionally confine LiPSs. In addition, microscopic probe techniques, such as in situ Kelvin probe force microscopy (KPFM), can be used to directly measure the change in the potential distribution at the heterogeneous interface during charging and discharging, which may establish the quantitative relationship between the charge gradient and polysulfide conversion efficiency.
(3) The expansion of MXene chemical diversity should be undertaken. MXenes, as a large family of 2D materials, have over 100 stoichiometric phases. Yet, only about 30 kinds of MXenes have been successfully synthesized to date, with most research focusing on Ti-based MXenes. Given the vast number of MXene family members and their tunable termination properties, this presents vast and limitless opportunities for realizing high-performance Li-S batteries. Therefore, exploring new MXene species with enhanced stability and electrochemical properties is a promising and intriguing research direction.
(4) Scalable manufacturing and economic feasibility should be addressed. Laboratory-scale MXene synthesis (e.g., HF etching) and coating processes (e.g., vacuum filtration) face severe challenges in cost control and industrial compatibility, particularly in achieving ultra-thin but defect-free MXene coatings required for high-energy-density batteries. For the synthesis of low-cost MXenes, developing Lewis acid salts or molten salts as substitutes for HF not only aligns with the principles of green chemistry but also helps reduce production costs. It is also possible to recycle MXene-based materials from waste separators, improving material recycling. For the low-cost industrialization of MXene-based hybrid separators with ultra-thin coatings, one could attempt to develop a 2D MXene self-assembled monolayer deposition process, which would greatly reduce the loading while still effectively blocking polysulfides. Meanwhile, in combination with the roll-to-roll manufacturing technique, it is possible to develop a coating process suitable for the large-scale production of MXene-based separators with ultra-thin coatings.

Overall, this review sought to emphasize the great advantages of MXene-based heterostructures for Li-S battery separator modification, extracting the general principles of heterostructure design, identifying the specific challenges, and suggesting possible research directions. This provides strong guidance for Li-S battery separator modification based on MXene-based heterostructures, offering a unique contribution.

## Figures and Tables

**Table 1:** Comparison of MXene-based heterostructures with a single MXene and other traditional materials for Li-S battery separator strategies.

**Materials**	**Core Advantages**	**Main Disadvantages**
Carbon	High conductivity Large surface area	Non-polar surfaces Brittle structure
Metal compound	Polar active sites Metal catalytic sites	Poor conductivity Low porosity Pulverization caused by volume expansion
Polymer	Robust flexibility Chemical functional groups	Poor conductivity Poor mechanical strength
MXene	Tunable terminations High conductivity Large surface area	Self-restacking Easily oxidized
MXene-based heterostructure	Introduction of built-in electric field Adjustment of the coordination environment Stable interface (hindering MXene oxidation and stacking) Ultra-high conductivity Synergistic adsorption–catalysis	

**Table 2:** Summary of electrochemical performance of MXene/inorganic material heterostructured hybrid separators for Li-S batteries.

	Heterostructures	Coating/Loading (mg cm^−2^)	Sulfur Loading/mg cm^−2^	Rate Capacity/Rate	Initial Capacity (mAh g^−1^)/Rate	Decay Rate/Cycles	Ref.
MXene/metal sulfide	NiS_2_/Ti_3_C_2_T_x_	Blade/0.8	1.07	921/2 C	1129/2C	0.038%/1000	[51]
	N-MX-CoS_2_	Filtration/0.2	1.2	775/4C	1031/1C	0.052%/700	[56]
	IL-MoS_2_/MX	Filtration/-	1.2–1.5	745.4/1C	764.4/1C	0.059%/700	[57]
	MXene@WS_2_	Filtration/0.6	1	622.2/3C	889.1/2C	0.0286%/2000	[58]
	1T-VS_2_/V_2_C			818.2/5 C	1131/1C	0.056%/1000	[59]
	VS_4_/Ti_3_C_2_T_x_	Filtration/0.15	1.2–1.5	673/4C	932/1C	0.059%/500	[60]
	ZnS/MXene	Blade/-	1	551/5C	810/0.5C	0.082%/500	[61]
	Co_1−x_S/3D-Ti_3_C_2_T_x_	Filtration/0.15	1.2–1.5		1056/1C	0.05%/500	[62]
	ZnS/MoS_2_@MXene	-/0.5	1	802.6/2C	900/1C	0.067%/500	[63]
	Bi_2_S_3_/MoS_2_@MX		0.8–1.2	592/5C	847/2C	0.069%/500	[31]
	MXene/MoS_2_/SnS@C	-/0.5	1	693.4/4C	~800/2C	0.051%/1000	[64]
	MXene/NiS_2_/Co_3_S_4_	Filtration/0.8	1	549.0/6C	956.3/2C	0.026%/2000	[65]
MXene/metal selenide	Ti_3_C_2_T_x_@CoSe_2_	Filtration/0.5	1.2	694/3C	1032.7/0.5C	0.059%/800	[53]
	Fe_3_Se_4_/FeSe@MXene	Blade/0.27	1.2	768.5/4C	~975/2C	0.07%/600	[69]
	MXene/Fe_3_S_4_@FeSe_2_	Filtration/0.8	1	557.1/5C	~825/2C	0.049%/1000	[70]
MXene/other inorganic metal	M-HTO-0.5	Blade/0.3–0.4	1.8–2.0	822.7/2C	~920/5C	0.073%/500	[72]
	Ta_4_C_3_-Ta_2_O_5_	-/0.9	1.0–1.5	738.5/1C	801.9/1C	0.086%/500	[54]
	Co_0.5_Ni_0.5_Te_2_/MXene	Blade/0.27–0.30	1.5	773/2C	~950/1C	0.0227%/500	[74]
	Ti_3_C_2_/CPNC	Blade/0.5	1.5	525.4/5C	928.4/1C	0.039%/1150	[75]
	Co_x_P@Ti_3_C_2_T_x_	Blade/-		751.61/3C	813.33/2C	0.037%/400	[76]
	Co_2_B@MXene	Filtration/-	1.2–1.5	597/5C	786/2C	0.0088%/2000	[77]
	MX@WSSe	Filtration/-	1.2–1.5	504.4/5C	600.2/2C	0.016%/1000	[78]
	Vo-LDHs-MXenes	Blade/-	2	342.9/3C	938.9/1C	0.084%/300	[52]
MXene/inorganic non-metal	g-C_3_N_4_/MXene	Coating machine/0.18	1.5	945/4C	858/1C	0.035%/1000	[83]
	BN@Mxene	Filtration/0.06	1.5–2	748/1C	686.9/1C	0.058%/700	[84]

**Table 3:** Summary of electrochemical performances of MXene/organic framework and polymer heterostructured hybrid separators for Li-S batteries.

Heterostructure	Coating/Loading (mg cm^−2^)	Sulfur Loading/mg cm^−2^	Rate Capacity/Rate	Initial Capacity (mAh g^−1^)/Rate	Decay Rate/Cycles	Ref.
MXene@COF		1.2–1.5	563/3C	~1050/0.5C	0.085%/200	[89]
MXene@TBCOF	-/0.29–0.31	1.65	493/5C	952.2/1C	0.0191%/1500	[32]
Ti_3_C_2_@iCON	Filtration/0.1	1.2	687/5C	810/2C	0.006%/2000	[90]
Ti_3_C_2_T_x_@Cu/Fe-MOF	Filtration/-	4.1	728/983/1C	1300/1400/1C	0.051%/0.064%/300	[93]
MXene/ANF	Filtration/-	1.0–1.5	818/5C	~975/3C	0.013%/3500	[94]
MXene-Nafion	Filtration/0.2	2	794/3C	920/1C	0.030%/1000	[95]
PA5-COOH/Nb_2_C	Filtration/-	1	303/10C	754/1C	0.047%/500	[96]

**Table 4:** Summary of electrochemical performance of MXene/carbon heterostructured hybrid separators for Li-S batteries.

Heterostructure	Coating/Loading (mg cm^−2^)	Sulfur Loading/mg cm^−2^	Rate Capacity/Rate	Initial Capacity (mAh g^−1^)/Rate	Decay Rate/Cycles	Ref.
N-Ti_3_C_2_/C	Blade/0.6	3.4	675/2C	1101.54/0.5C	0.07%/500	[102]
CNVM	Blade/0.38	1.2	723/3C	867/1C	0.075%/660	[103]
Ti-N-Ti_3_C_2_Cl-C	-/0.12	1.0–1.5	518.6/4C	761/2C	0.053%/500	[104]
DNA-CNT/MXene	Filtration/0.1	1	588/2C	798/1C	0.13%/200	[55]
CNTs/MXene	Filtration/0.16	0.8	728/2C	987/1C	0.06%/600	[105]
Ti_3_C_2_T_x_/CNTs 10%	Filtration/0.016	1.2	640/2C	760/1C	0.086%/200	[106]

## Data Availability

No new data were created or analyzed in this study. Data sharing is not applicable to this article.
